# PREDICTING FALLS IN OLDER ADULTS

**DOI:** 10.1093/geroni/igad104.2280

**Published:** 2023-12-21

**Authors:** Colleen Brown, Marina Oktapodas Feiler, Eleanor Simonsick

**Affiliations:** Temple University, Philadelphia, Pennsylvania, United States; Temple University, Philadelphia, Pennsylvania, United States; NIA IRP, Baltimore, Maryland, United States

## Abstract

Falls are detrimental to older adults causing injury and mortality. Although clinical assessments of balance deficits exist, it is not well understood how well they predict falls. Longitudinal analysis utilized data from 688 men and women aged 60-97 enrolled in the Baltimore Longitudinal Study of Aging with index and subsequent visits approximately 1.7 years apart. Inclusion criteria included objective balance assessment at index visit and answering the question “Have you fallen in the past 12 months?” at both visits. Predictors of interest included thigh muscle strength, progressive static balance test performance, narrow walk, and sway reference with eyes closed/feet together. Tests were parameterized using validated methods. Adjusted logistic regression was modeled to estimate the association between objective measures of balance and fall risk. Adjustment variables included age, sex, race, height, and weight. Results reported those who failed the eyes closed/feet together position had 3.6 (95% CI 1.03, 12.81) times the odds of future falls compared with those who passed. Those who failed the semi-tandem, tandem, and single leg stance, had respectively 3.1 (1.44, 6.75), 3.6 (1.59, 8.06) and 4.8 (1.93, 12.09) times greater odds of history of falls compared with those who did not fail. Progressive standing balance performance did not predict future falls. The eyes closed/feet together test is easily administered, requires no equipment, and takes less than 30 seconds to complete. Failure of eyes closed/feet together may predict future falls, which is clinically relevant as this may suggest shifting the momentum towards proactively preventing an initial fall.

